# A Mobile Health Intervention for Prostate Biopsy Patients Reduces Appointment Cancellations: Cohort Study

**DOI:** 10.2196/14094

**Published:** 2019-06-02

**Authors:** Ashwin S Balakrishnan, Hao G Nguyen, Katsuto Shinohara, Reuben Au Yeung, Peter R Carroll, Anobel Y Odisho

**Affiliations:** 1 Department of Urology University of California San Francisco San Francisco, CA United States; 2 Helen Diller Family Comprehensive Cancer Center San Francisco, CA United States

**Keywords:** text messaging, appointments and schedules, mHealth, quality improvement, urology, prostate neoplasm

## Abstract

**Background:**

Inadequate patient education and preparation for office-based procedures often leads to delayed care, poor patient satisfaction, and increased costs to the health care system. We developed and deployed a mobile health (mHealth) reminder and education program for patients scheduled for transrectal prostate biopsy.

**Objective:**

We aimed to evaluate the impact of an mHealth reminder and education program on appointment cancellation rates, communication frequency, and patient satisfaction.

**Methods:**

We developed a text message (SMS, short message service)–based program with seven reminders containing links to Web-based content and surveys sent over an 18-day period (14 days before through 3 days after prostate biopsy). Messages contained educational content, reminders, and readiness questionnaires. Demographic information, appointment cancellations or change data, and patient/provider communication events were collected for 6 months before and after launching the intervention. Patient satisfaction was evaluated in the postintervention cohort.

**Results:**

The preintervention (n=473) and postintervention (n=359) cohorts were composed of men of similar median age and racial/ethnic distribution living a similar distance from clinic. The postintervention cohort had significantly fewer canceled or rescheduled appointments (33.8% vs 21.2%, *P*<.001) and fewer same-day cancellations (3.8% vs 0.5%, *P*<.001). There was a significant increase in preprocedural telephone calls (0.6 vs 0.8 calls per patient, *P*=.02) in the postintervention cohort, but not a detectable change in postprocedural calls. The mean satisfaction with the program was 4.5 out of 5 (SD 0.9).

**Conclusions:**

An mHealth periprocedural outreach program significantly lowered appointment cancellation and rescheduling and was associated with high patient satisfaction scores with a slight increase in preprocedural telephone calls. This led to fewer underused procedure appointments and high patient satisfaction.

## Introduction

### Background

Surgical procedures are increasingly being performed in outpatient facilities such as physician offices or ambulatory care centers [1]. From 1981 to 2009, outpatient procedure volume grew from approximately 110,000 to 12 million procedures [2]. At least 60% of urologic procedures are performed in the outpatient setting, with prostate biopsy and cystoscopy being the most common outpatient urologic procedures [3]. Prostate biopsy remains essential for diagnosing and monitoring prostate cancer. However, safe in-office biopsy requires preprocedural preparation, such as prophylactic antibiotics, and postprocedure symptom assessment. Inadequate education regarding the importance of prostate biopsy and preparation for the procedure can lead to delays in care, canceled appointments, decreased patient satisfaction, and costs to the health care system [4,5]. Prior reports have found that clinics can lose up to 16.4% of their daily potential revenue because of late cancellations or no-shows [6,7]. Moreover, patients often have anxiety about discomfort they may experience, procedure-related risks such as infection, and receiving concerning biopsy results, which likely contribute to missed appointments and nonadherence to patient instructions [8-10].

Health systems are expanding their use of mobile technologies to improve communication with patients. Several trials have shown that text message (short message service, SMS) reminders can improve medication adherence and clinical attendance for appointments [11,12]. Mobile health (mHealth) interventions have also led to improved rates of patient adherence in colon, breast, and cervical cancer surveillance cohorts [13-15]. In prostate cancer care, electronic health (eHealth) and mHealth interventions have helped patients understand their disease severity, weigh the risks and benefits of various treatment options, and track key information such as prostate-specific antigen (PSA) laboratory results [16]. However, there are no mHealth interventions in urology that address the actual receipt of care and the interaction between patients, providers, and the clinic [16]. Prostate biopsies are a relatively complex patient encounter that provides an opportunity to improve communication and efficient care delivery with an mHealth intervention. In order to undergo a safe prostate biopsy, patients may need to hold anticoagulation medications prior to the procedure, take prophylactic antibiotics at home, self-administer a rectal enema, or get preprocedural magnetic resonance imaging (MRI). After the procedure, patients are at risk for bleeding and infection.

### Objectives

We developed and deployed an mHealth SMS-based reminder, education, and procedure preparedness assessment program for patients scheduled for transrectal prostate biopsy and evaluated the impact on patient appointment completion. We hypothesized that rates of canceled or rescheduled appointments would decrease following deployment of the program. We also evaluated communication frequency between patients and providers to assess for potential changes in provider workloads and patient satisfaction with the mHealth intervention following implementation of the program.

## Methods

### Development

We developed an SMS-based program for patients undergoing MRI–transrectal ultrasound (TRUS) fusion prostate biopsy at a busy academic urologic oncology practice at the University of California, San Francisco (UCSF). The program consisted of 8 text messages sent over an 18-day period, with the first message sent 14 days before prostate biopsy and the last message sent 3 days after the procedure (Table 1). The text messages contained short reminders or educational material with links to more detailed Web-based content and short questionnaires (2 to 4 questions) that could be viewed on a mobile phone. Educational content included step-by-step descriptions of the biopsy procedure, the importance of antibiotic and enema adherence, and an embedded animated video on the importance of getting a prostate biopsy. If patients had a mobile phone without internet capabilities, they could still view the text messages but did not have access to linked content. We developed software that integrated with the electronic health record (EHR; Epic Systems Corporation) to extract demographic data and contact information for a patient when they are scheduled for a prostate biopsy and automatically enroll them to receive text messages. We used services from a commercial provider (Medumo Inc) to send text messages and log results. Patients received a short message at the time of appointment scheduling which welcomed them to the program and allowed them to opt out of receiving messages.

We employed a development process detailed in an earlier report to prototype, refine, and evaluate the SMS-based intervention [17]. We first collated all materials given to patients in clinic or via the EHR patient portal and call scripts for periprocedural reminder phone calls. We engaged stakeholders (clinic managers, nurses, and urologists) to identify key prostate cancer concepts that patients expressed difficulty understanding and the nature and timing of preparatory steps that patients had difficulty following. With the guidance of clinical staff and providers, preparatory instructions and patient education concepts within all materials were identified, modified, and incorporated in the mHealth intervention. These included instructions and questionnaires for antibiotic adherence, anticoagulation management, enema use, and confirmation of completion of prostate MRI for patients undergoing MRI-fusion biopsy. Postprocedure symptoms were assessed via questionnaire. Messages and Web-based content were written to an 8th grade reading level using the Flesch-Kincaid Grade Level [18].

Any concerning preprocedure responses (such as failing to stop anticoagulation) triggered an email to the clinic manager, who then triaged follow-up to the appropriate nurse. Concerning postprocedure responses (fever, bleeding) prompted patients to contact the clinic (routed to the on-call physician after hours) in addition to triggering an email alert. Informed by the expertise of clinical providers and the American Urological Association guidelines for prostate biopsy, we identified the ideal temporality of content to guide when text messages should be sent to patients (Table 1) [19].

**Table 1:** Schedule of text messages sent to patients.

Day	Time	Content sent
At registration	Enrollment	Program welcome, patient homepage link
14 days before	10 am	Magnetic resonance imaging and medication survey
12 days before	10 am	Educational information and video on program
7 days before	9 am	Key items to obtain and fleet enema instructions
1 day before	9 am	Preprocedure readiness survey
Day of procedure	7 am	Antibiotic and Fleet enema reminder
Day of procedure	5 pm	Postprocedure precautions
2 days after	10 am	Follow-up symptom survey
4 days after	5 pm	Satisfaction survey

### Study Design and Data Sources

The program was launched on May 1, 2018, as a practice-wide quality improvement initiative. The preintervention cohort was defined as patients undergoing prostate biopsy in the 6-month period prior to program launch (November 1, 2017, to April 30, 2018) and the postintervention cohort was defined as patients undergoing prostate biopsy during a 6-month period (June 1 to November 30, 2018); patients were required to have a phone number to a mobile phone with SMS capabilities stored in our medical record system. There was a 1-month washout period dividing the two cohorts to account for patients who had biopsies scheduled within 14 days and would therefore not receive the full sequence of text messages. We retrospectively obtained appointment and communication frequency statistics.

Demographic information (age, race/ethnicity, distance from home to our clinic [km], urban vs rural geography, Diez-Roux neighborhood score based on patient zip code as a proxy for socioeconomic status [20], and insurance type), data on the occurrence and timing of appointment cancellations and rescheduling, and patient-provider communications (patient-provider phone calls and Epic MyChart in-basket messages) were collected from the electronic medical record for patients in the preintervention and postintervention cohorts. Providers involved with patient communication included urologists, nurse practitioners, registered nurses, and nurse navigators.

### Outcomes and Analyses

The primary outcome of this study was the percentage of prostate biopsy appointments that were canceled or rescheduled. We further categorized cancellations by their temporality (same-day or within 7 and 14 days of scheduled appointment). We defined cancellation lead time as the number of days before the scheduled appointment that the appointment was canceled or rescheduled. Secondary outcomes included nature and frequency of patient-provider communications and patient satisfaction. Communications were defined as being preprocedural (within 14 days before appointment), postprocedural (within 7 days after), or periprocedural (14 days before to 7 days after). In-basket messages were defined as all relevant patient-provider and provider-provider messages in the Epic MyChart portal. Patient messages to providers regarding medical advice, provider messages to patients, and provider-provider messages were also collected and categorized. Patient satisfaction, as gauged by three survey questions delivered via text message on the last day of the program, was evaluated in the postintervention cohort. The questions were scored on a 5-point Likert scale and included the following: (1) How highly would you recommend this digital instruction program to a family member or friend? (1 = would not recommend and 5 = would highly recommend), (2) Overall, how satisfied are you with the care you received? (1 = not satisfied at all and 5 = very satisfied), and (3) What did you think of the number of reminders? (1 = far too many messages, 3 = the right number of messages, and 5 = far too few messages).

Baseline differences in the preintervention and postintervention cohorts were compared using chi-square tests for categorical factors and sample *t* tests or Mann-Whitney tests for continuous factors. All analyses were performed using R 3.5.1 (The R Foundation). This study was approved by the UCSF institutional review board.

## Results

### Sample Characteristics

**Figure 1:**
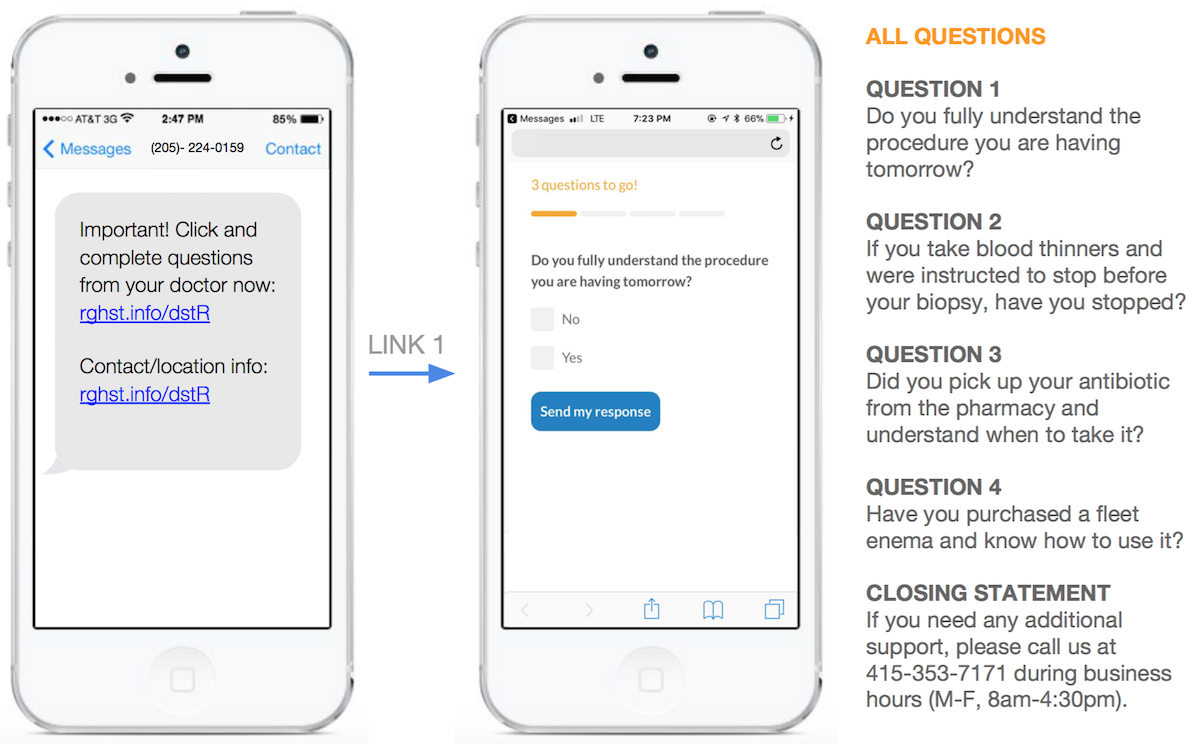
Text message with link to Web-based questionnaire sent to patients the day before procedure.

There were 473 patients in the preintervention cohort (November 1, 2017, to April 30, 2018) who did not receive the SMS program, and 359 patients in the postintervention cohort who were enrolled in the 18-day mHealth program (Table 1 and Figure 1). Four eligible patients (1.1%) in the postintervention cohort opted out of the mHealth program. The preintervention and postintervention cohorts were composed of patients of similar median age (67.0 vs 67.6 years, *P*=.55) and of comparable racial/ethnic demographics (75.3% vs 76.0% white, *P*=.44; Table 2). Patients in both cohorts lived a similar median distance from care (74 vs 73 km, *P*=.74) and primarily lived in urban or metropolitan areas (88.5% vs 87.1%, *P*=.68). There were no differences in socioeconomic status as measured by neighborhood score (*P*=.39).

**Table 2:** Patient demographics in the preintervention and postintervention cohorts.

Characteristic		Preintervention (n=473)	Postintervention (n=359)	*P* value
Age in years, median (IQR^a^)		67.5 (61.9-72.2)	67.0 (61.3-72.5)	.55
**Ethnicity, n (%)**				.44
	Caucasian	356 (75.3)	273 (76.0)	
	Black or African American	30 (6.4)	18 (5.0)	
	Hispanic or Latino	14 (2.9)	17 (4.8)	
	Asian	28 (5.9)	21 (6.0)	
	Other/unknown	45 (9.6)	30 (8.3)	
Distance from clinic (km), median (IQR)		74 (21-202)	73 (23-192)	.74
Urban, n (%)		419 (88.5)	313 (87.1)	.68
Rural, n (%)		53 (11.2)	43 (12.1)	
Neighborhood score, median (IQR)		4.6 (1.7-7.5)	3.9 (1.3-7.5)	.39
**Insurance, n (%)**				.88
	Commercial insurance	187 (39.6)	148 (41.2)	
	Medicare	266 (56.3)	194 (54.0)	
	Medi-Cal	18 (3.8)	15 (4.3)	
	Self-pay	2 (0.3)	2 (0.5)	

^a^IQR: interquartile range.

### Appointment Cancellation or Rescheduling

There were 37.3% fewer canceled or rescheduled appointments in the postintervention cohort compared with the preintervention cohort (33.8% vs 21.2%, *P*<.001; Table 3). Same-day cancellations were reduced by 86.8% with the intervention, with 3.8% of patients in preintervention cohort canceling on the day of their appointment compared with 0.5% in postintervention cohort (*P*<.001). Appointment cancellation or rescheduling within 7 days (13.1% vs 8.6%, *P*=.03) and within 14 days (19.0% vs 12.6%, *P*=.01) was also significantly lower in the postintervention cohort. There was not a detectable difference in the median lead time to cancellation or rescheduling between the preintervention and postintervention cohorts (10.7 vs 10.8 days, *P*=.32). There were no detectable differences in patient age, race/ethnicity, geography, neighborhood socioeconomics, or insurance type between patients canceling or rescheduling appointments and those who did not.

### Patient-Provider Communication

Compared with the preintervention period, in the postintervention period there was a significant increase in preprocedural in-basket messages (3.6 vs 4.1 messages per patient, *P*=.04) but not in postprocedural in-basket messages (1.3 vs 1.4 messages per patient, *P*=.56; Table 4). There were no detectable differences in periprocedural messages from patients to providers (1.6 vs 1.8, *P*=.34) or messages from providers to patients (1.5 vs 1.5, *P*=.88) when comparing the preintervention and postintervention cohorts. When looking at provider-provider in-basket message communication, there were significant increases in message volumes forwarding patient charts with comments (0.5 vs 0.9, *P*<.001) and clinic orders (0.6 vs 0.9, *P*<.006) in the postintervention period. After launching the intervention, there was an increase in preprocedural phone communication with patients (0.6 vs 0.8 telephone calls per patient, *P*=.02) but not in postprocedural phone communications.

**Table 3:** Appointment cancellation and rescheduling in the preintervention and postintervention cohorts.

Characteristic			Preintervention (n=627^a^)		Postintervention (n=420^a^)		*P* value
Total patients, n			473		359		
Appts^b^, completed, n (%)			412 (65.7)		330 (78.6)		<.001
Appts, no-show, n (%)			3 (0.5)		1 (0.2)		.92
**Appts, canceled or rescheduled, n (%)**			212 (33.8)		89 (21.2)		<.001
	Canceled or rescheduled, same-day	24 (3.8)		2 (0.5)		<.001	
	Canceled or rescheduled, within 7 days	85 (13.1)		38 (8.6)		.03	
	Canceled or rescheduled, within 14 days	119 (19.0)		54 (12.6)		.01	
Cancel or reschedule lead days, median (IQR)^c^			10.67 (2.86-24.86)		10.79 (4.75-27.92)		.32

^a^All percentages are reported as a proportion of the total appointments scheduled in each column.

^b^Appts: appointments.

^c^IQR: interquartile range.

**Table 4:** Periprocedural communication volume per patient in preintervention and postintervention periods.

Characteristic		Preintervention, mean (SD)^a^	Postintervention, mean (SD)^a^	*P* value
**All in-basket messages**		4.9 (5.0)	5.6 (5.6)	.05
	Preprocedural	3.6 (4.0)	4.1 (4.9)	.04
	Postprocedural	1.3 (2.3)	1.4 (2.3)	.56
**Patient message to provider**		1.6 (2.6)	1.8 (3.0)	.34
	Preprocedural	1.1 (2.0)	1.3 (2.4)	.11
	Postprocedural	0.5 (1.3)	0.4 (1.2)	.46
**Provider message to patient**		1.5 (1.8)	1.5 (2.2)	.88
	Preprocedural	1.2 (1.5)	1.2 (2.1)	.87
	Postprocedural	0.3 (0.8)	0.3 (0.8)	.42
**Provider-provider message**		2.1 (2.4)	2.5 (2.2)	<.001
	Preprocedural	1.5 (1.9)	1.7 (1.7)	.05
	Postprocedural	0.6 (1.1)	0.8 (1.1)	.01
**Patient-provider telephone call**		0.8 (1.1)	1.0 (1.6)	<.001
	Preprocedural	0.6 (1.0)	0.8 (1.5)	.02
	Postprocedural	0.2 (0.5)	0.2 (0.5)	.24

^a^All values represent mean (SD) messages per patient, per appointment.

### Patient Satisfaction

Mean patient satisfaction with the text message program was 4.5 out of 5 (SD 0.9), and the mean satisfaction with overall care was 4.8 out of 5 (SD 0.6, see Methods section for details on questions and scoring system). Patient opinion of text message quantity was 2.8 out 5, with a score of 3 corresponding to the right number of messages (SD 0.4).

## Discussion

### Principal Findings

An SMS-based mHealth periprocedural outreach program significantly lowered both last-minute and overall appointment cancellation and rescheduling and was associated with high patient satisfaction scores and a low opt-out rate. While the number of secure message and telephone interactions with patients slightly increased, this was associated with fewer underused procedure appointments and high patient satisfaction.

### Preventing Appointment Cancellations

Appointment cancellations, particularly same-day cancellations and no-shows, can significantly burden health care systems. Additionally, inability to undergo the procedure after arrival due to inadequate preprocedure preparation is a significant time burden and inconvenience to patients, who often drive long distances from their home or take time off from work. For patients being evaluated for prostate cancer, procedure completion is essential for providing timely care. A meta-analysis of randomized controlled trials assessing the impact of SMS reminders in a wide range of practice settings found that reminders significantly increase attendance to health care appointments [12]. These results have been reinforced in the cancer screening literature. A systematic review of the impact of text message interventions on cancer screening rates found that absolute screening rates for patients receiving SMS reminders was 0.6% to 15% higher than for controls [14]. Cancer screening or surveillance patients sometimes share similar needs in terms of procedural preparation and patient education. For example, patients undergoing workup for colon cancer and prostate cancer both require adherence to several preparatory steps. In a randomized trial, Deng and colleagues found that an SMS reminder program significantly reduced cancellations from 8.0% to 4.8% in a clinic performing gastrointestinal endoscopy under sedation [13]. Similar to our program for patients undergoing prostate biopsy, their program contained reminders for key preparatory steps that often lead to cancellation, such as failure to discontinue an anticoagulant.

### Selecting the Appropriate Platform

As more technologies for patient-provider communication become available, it is important to choose the appropriate method of communication to match the needs and technology literacies of specific patient populations. Web- and SMS-based technology has shown considerable promise in improving care for patients with prostate cancer. Kenfield and colleagues [21] found that patients with prostate cancer are amenable to using digital interventions (interactive website, text messaging, and a physical activity tracker) and that these interventions helped them adopt recommended lifestyle and dietary changes. A mobile phone app developed for detection and management of symptoms during prostate cancer treatment was found to reduce urinary-related symptoms and improve emotional functioning [22].

These earlier interventions for prostate cancer patients are designed for long-term care of patients while they undergo and recover from prostate cancer treatment. Patients are often highly invested in their preparation for surgery and therefore may be more willing to put in the effort to download a phone app or wear an activity tracking device. In comparison to surgery, prostate biopsy has a shorter periprocedural period and requires less physical and emotional preparation on the part of the patient. After considering several mobile app- or Web portal–based interventions, we decided to instead develop an SMS-based reminder program because this technology requires relatively less effort to engage with and has become widely adopted in our study population. Unlike mobile apps and Web portal–based interventions, SMS does not require the patient to download any software or create log-in credentials.

When deciding which patient communication platform to use, providers must assess the impact that any intervention will have on clinic workflow and capacity. We developed software in-house, which allowed us to automate enrollment in the text program. Practices that are not able to do this may need clinic staff to manually enroll patients in the program or rely on a commercial provider to help with this process. We also designed the program to trigger alerts to our clinic manager when a patient had a concerning response to a survey question. This also adds work for the clinical team. If a practice does not have capacity to manage these alerts, the survey questions and/or alerts can be removed. The program will still provide valuable reminders and educational information to patients without the triggered alerts.

### Tailoring Interventions

Since reasons for cancellation often vary based on the type of appointment and patient population, customized interventions are needed to prevent cancellations and improve preparation [23]. Rather than simply reminding patients about their appointment, our intervention was customized to include educational information and survey questions specific to prostate biopsy patients. The study by O’Dwyer et al [23] of canceled elective urology appointments found that the majority of procedure-room cases (prostate biopsy, cystoscopy, and catheter changes) were canceled because patients were not adequately prepared for surgery. A study of procedural cancellations in a pediatric urology clinic found that many cancellations were due to preventable factors such as fasting violations [24]. These investigations reinforce the need for tailored interventions that address the specific needs of certain patient populations and complement the work of clinic staff rather than one-size-fits-all reminder systems.

### Communication Volume

Patient communications with providers via telephone calls in the preprocedural period increased, while telephone calls in the postprocedural period did not significantly change. While the effect size of the preprocedural increase in call volume was small, it is possible that it created meaningful changes in clinic workloads. Provider-provider communication also increased significantly, which may be due to increased communication about how to manage patient concerns reported over telephone or concerning responses to survey questions in the intervention. Increases in periprocedural communication may be necessary in order to adequately prepare patients for prostate biopsy, avoid scheduling inefficiencies, and prevent patient complaints that would lead to increased postprocedural communications.

### Limitations

This study has limitations. Although we were able to track which patients clicked links in the text messages and opened Web-based content, for patients who did not click the links in the SMS, there is no other way with current SMS technology to assess if they received or read the message. This means that we are likely undercounting the degree of engagement, which biases our results toward the null. Patients were not directly engaged as stakeholders in the intervention development process. However, the program was based on materials given to patients in clinic that patients extensively helped to develop. We are currently gathering patient feedback on the program, which will directly inform future interventions. Although we aimed for an English 8th grade reading level, the intervention was not translated into other languages. Future interventions can assess patient language preferences and deliver language appropriate programs. The ethnic and racial composition of our cohort did not match the composition of the United States as a whole or the surrounding region, which may impact generalizability. Moreover, the majority of patients lived in urban areas, which likely impacts the generalizability of results to rural populations. Prior studies have found that SMS-based interventions have helped to improve clinical attendance and/or engage underserved populations in cancer screening and educational efforts [15,25,26]. Therefore, we aim to increase this proportion in future research by including clinical sites with greater proportions or racial/ethnic minorities and other vulnerable populations. However, there were no detectable demographic or socioeconomic differences between the preintervention and postintervention cohorts. Some patient-provider communications are not appropriately documented in the EHR. For example, communication with nonprovider clinic staff was not captured in our medical record and therefore not analyzed in this study. As an observational study, it is subject to selection bias. However, the study included six months of patient appointments in both the preintervention and postintervention cohorts, which may mitigate potential biases caused by short-term secular trends.

This study also has several strengths. First, we leveraged appointment scheduling logs in the EHR to obtain detailed information on the occurrence and timing of appointment cancellation or changes. Second, compared with other SMS-based interventions which require manual enrollment of patients, our intervention had an otherwise lower impact on clinic staff workloads as enrollment was automated and did not require any changes to workflow. Moreover, we were able to assess changes that the SMS program may have on clinic workloads by extracting and analyzing data on the type and frequency of patient-provider and provider-provider communications. Last, we considered the reading proficiency and technology literacy of our patient population in the design of the program.

### Conclusions

An mHealth periprocedural outreach and reminder program designed specifically for prostate biopsy patients significantly lowered appointment cancellations and was associated with high patient satisfaction scores. The number of secure message and telephone calls per patient increased slightly; however, this increase in communication may be necessary in order to improve clinic efficiency and patient satisfaction. Future research on predictors of engagement with periprocedural SMS-based interventions and the impact of these programs with diverse study populations will help to understand the utility of this intervention among different patient groups.

## Abbreviations

EHR: electronic health record
IQR: interquartile range
MRI: magnetic resonance imaging
PSA: prostate-specific antigen
SMS: short message service
TRUS: transrectal ultrasound
UCSF: University of California, San Francisco
